# Bone marrow fat change in pediatric patients with non-alcoholic fatty liver disease

**DOI:** 10.1371/journal.pone.0234096

**Published:** 2020-06-02

**Authors:** Salman S. Albakheet, Haesung Yoon, Hyun Joo Shin, Hong Koh, Seung Kim, Mi-Jung Lee

**Affiliations:** 1 Department of Radiology, Severance Hospital, Severance Pediatric Liver Disease Research Group, Research Institute of Radiological Science, Yonsei University College of Medicine, Seoul, South Korea; 2 Department of Radiology, King Faisal General Hospital, Al-Hofuf, Kingdom of Saudi Arabia; 3 Division of Gastroenterology, Hepatology and Nutrition, Department of Pediatrics, Severance Children’s Hospital, Severance Pediatric Liver Disease Research Group, Yonsei University College of Medicine, Seoul, South Korea; Auburn University, UNITED STATES

## Abstract

**Objectives:**

To investigate changes of fat in bone marrow (BM) and paraspinal muscle (PSM) associated with the degree of fatty liver in pediatric patients with non-alcoholic fatty liver disease (NAFLD) in consideration of age and body mass index (BMI).

**Methods:**

Hepatic fat, BM fat, and PSM fat from proton density fat fraction of liver MRI between June 2015 and April 2019 were quantitatively evaluated on axial images of the fat map at the mid-level of T11-L2 vertebral bodies for BM fat and at the mid-level of L2 for PSM fat. Age, height, and weight at the time of MRI were recorded and BMI was calculated. Correlation analysis was performed.

**Results:**

A total of 147 patients (114 male) were included with a mean age of 13.3 ± 2.9 years (range 7–18 years). The mean fat fractions were 24.3 ± 13.0% (2–53%) in liver, 37.4 ± 8.6% (17.3–56%) in vertebral BM, and 2.7 ± 1.1% (1.0–6.9%) in PSM. Age, height, weight, and BMI were not correlated with liver fat or BM fat. However, weight (ρ = 0.174, p = 0.035) and BMI (ρ = 0.247, p = 0.003) were positively correlated with PSM fat. Liver fat showed positive correlation with BM fat when adjusting age and BMI (ρ = 0.309, p<0.001), but not with PSM fat.

**Conclusions:**

BM fat positively correlates with liver fat, but not with age or BMI in pediatric NAFLD patients.

## Introduction

With sedentary lifestyle there is a considerable increase in the prevalence of obesity among pediatric and adolescent children with an estimated prevalence in developed countries greater than 30% [1]. The World Health Organization summarized that worldwide obesity has nearly tripled since 1975 and over 340 million children and adolescents aged 5–19 were overweight or obese in 2016. Accompanied with increased obesity, non-alcoholic fatty liver disease (NAFLD) has increased in children and is considered as the most common liver disease among pediatric patients in the developed world [2–4]. However, there is great variation in the reported prevalence of NAFLD in children with obesity, with a wide range of prevalence from 1.7% to 85% [5–7]. This variability in prevalence estimates could be due to different body mass index (BMI) references, study design, ethnicity, and geography [7].

Pediatric NAFLD can cause not only chronic liver disease but also extrahepatic complications such as cardiovascular disease, dyslipidemia, type 2 diabetes mellitus, hypertension, and low bone mineral density [8–10]. The main organ for lipid metabolism is the liver; therefore, insulin resistance in liver and adipose tissue in obese patients can cause excessive free fatty acids and the subsequent accumulation of triglycerides in hepatocytes and other tissues including skeletal muscle and bone marrow (BM) [11–13].

Obese children with NAFLD are more susceptible to low bone mineral density than children without obesity or NAFLD [14,15]. However, there are limited studies about BM fat content in pediatric NAFLD patients, and the published studies report discrepant results [16,17]. In adolescents, hematopoietic marrow is progressively converted to fatty marrow. Therefore, we need to differentiate normal fatty marrow conversion with abnormal BM fat infiltration. In addition, there are some reports about the correlation between fatty liver and peripheral muscle insulin sensitivity [18,19]. However, no reports have focused on the relationship between skeletal muscle fat content and fatty liver. Therefore, the purpose of this study was to investigate the changes of fat in BM and PSM associated with the degree of fatty liver in pediatric patients with known or suspected NAFLD in consideration of age and BMI.

## Materials and methods

### Study population

The approval of institutional review board of Severance Hospital was obtained for this retrospective study (approval number 4-2019-0394) for anonymized data review and the need to obtain informed consent was waived. This study included consecutive pediatric patients who underwent fat quantification liver MRI, including proton density fat fraction (PDFF), between June 2015 and April 2019 to evaluate NAFLD in our academic children’s hospital. All patients had known NAFLD or suspected NAFLD based on prior clinical history, ultrasonographic findings (increased liver parenchymal echo), or unexplained liver enzyme (alanine aminotransferase, aspartate aminotransferase, or γ-glutamyltransferase) elevation with obesity. We excluded patients who had clinical or laboratory evidence of liver disease other than NAFLD (e.g., glycogen storage disease, hepatic congestion from heart disease, or virus hepatitis) or hepatic mass. Patient age, height, and weight were recorded at the time of MRI examination and BMI was calculated. To evaluate the effect of obesity in our study population, we divided the patients into three groups based on BMI as normal, overweight and obesity. Overweight was defined as a BMI greater than the 85th percentile for age, and obesity was defined as a BMI at or above the 95th percentile for children of the same age and sex.

### Imaging study

All MRI examinations were performed with patients lying supine on a 3T MRI system (Discovery 750w, GE Healthcare, Waukesha, WI, USA) with a 32-channel body coil. MRI acquisition included single shot fast spin echo T2-weighted axial and coronal images and iterative decomposition of water and fat with echo asymmetry and least-squares estimation (IDEAL-IQ) axial images of the liver. Single shot fast spin echo images were used to identify anatomical locations of the liver, T11-L2 vertebral bodies, and PSM. The IDEAL-IQ sequence was used to obtain PDFF (water-triglyceride fat separation) maps of the abdomen from a single breath-hold acquisition. The parameters of IDEAL-IQ were as follows: repetition time, 5.8 msec; field of view, 35–42 cm; bandwidth, 125 kHz; flip angle, 3°; section thickness, 8 mm; and a single three-dimensional image with 25 to 30 sections.

### Image analysis

All images were retrospectively reviewed and analyzed by a musculoskeletal radiologist with 6 years of experience in musculoskeletal radiology who was blinded to the electronic medical records of the patients, including the radiology report and the final diagnosis. All images were digitally assessed using a commercially-available picture archiving and communications system workstation (Centricity® Radiology RA1000, GE Healthcare, Barrington, IL, USA). Hepatic PDFF measurements were performed by drawing regions of interest (ROIs) with the maximal area in the right hepatic lobe in three contiguous axial images. The ROIs were oval or circular in shape and excluded the liver boundary, fissures, gall bladder fossa, artifacts, and large blood vessels. The PDFF measurements of vertebral BM were performed by drawing four ROIs with the maximal area at the mid-level of the each T11, T12, L1, and L2 vertebral body. The ROIs excluded the endplates and vertebral boundaries. The PDFF measurements of PSM were also performed by drawing ROIs with the maximal axial area in PSM bilaterally at the mid-level of L2. The average values of liver, BM, and PSM fat fraction measurements were calculated and used as the representative value in each patient.

### Statistical analysis

All statistical analyses were performed using the SPSS software package (IBM SPSS Statistics version 21; IBM Corp., Armonk, NY, USA), SAS (version 9.4; SAS Inc., Cary, NC, USA) and PASS (version 12; NCSS, LLC, Kaysville, Utah, USA). Independent t-test was used for the comparison of gender and BMI groups. Pearson’s correlation coefficient (ρ) was calculated to evaluate the correlations between variables. Partial correlation analysis was also performed. In addition, we performed a linear regression analysis of the correlation variables including both simple and multiple linear regression in considering with interaction. The power of correlation analyses were verified using post-hoc power analysis. A p-value < 0.05 was deemed to indicate statistically significant differences.

## Results

### Patient characteristics and fat fractions

A total of 147 patients (114 male and 33 female) were included in this study. The mean age was 13.3 ± 2.9 years with a range of 7–18 years. The range of patient height was 100–186 cm and that of weight was 18–133 kg. The BMI of the patients ranged from 14.2–41.0 kg/m^2^ with a mean of 27.5 ± 5.2 kg/m^2^. Based on BMI, 32 patients were overweight and 92 patients were obese.

Among the patients, 135 patients had increased fat infiltration in the liver, and the remaining 12 patients (including three overweight and five obese patients) had a normal limit of fat as less than 6% [20] in liver based on PDFF. The mean hepatic fat fraction was 24.3 ± 13.0% with a range of 2–53%. The fat fraction could be obtained from all the T11-L2 vertebral levels for BM fat and the mid L2 levels for PSM fat in all included patients. The range of BM fat fraction was 17.3–56.0% with a mean of 37.4 ± 8.6%. The range of PSM fat fraction was 1.0–6.9% with a mean of 2.7 ± 1.1%.

There were only four patients (three boys and one girl) with normal BMI and no hepatic fat infiltration. The age range was 9–16 years, BM fat fraction was 28.2–52.0%, and PSM fat fraction was 1.1–2.5% in these four patients.

When comparing boys and girls, boys were taller (160.5 ± 14.6 cm vs. 153.6 ± 14.4 cm, p = 0.018) and heavier (73.9 ± 24.0 kg vs. 63.4 ± 19.3 kg, p = 0.012) than girls. However, age, BMI, liver fat, BM fat, and PSM fat were not different between the two groups.

In BMI group analysis, liver fat and BM fat were not different among the three groups. However, PSM fat was lower in the normal group (2.0 ± 0.8%) compared with overweight (2.8 ± 1.1%, p = 0.016) and obese (2.9 ± 1.1%, p = 0.001) groups.

### Correlation and regression results

**Fig 1:**
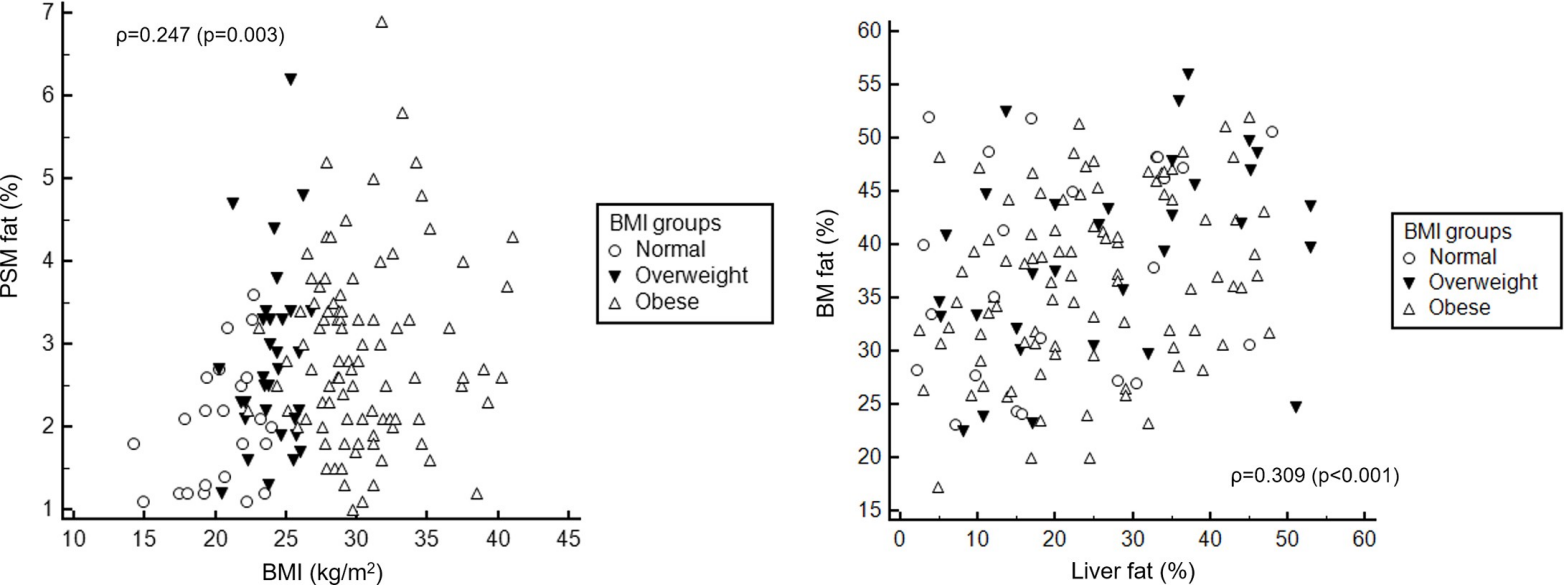
Scatter plots for the correlations between variables including body mass index (BMI) groups. Based on BMI, 23 patients were normal, 32 patients were overweight, and 92 patients were obese. (A) There was a poor, but positive correlation between BMI and paraspinal muscle (PSM) fat fraction (ρ = 0.247, p = 0.003). (B) There was also fair and positive correlation between liver and bone marrow (BM) fat fractions (ρ = 0.309, p<0.001). From the linear regression analysis, the slope (β) between BMI and PSM fat was 0.05 (R = 0.244, p = 0.003) and that between liver fat and BM fat was 0.20 (R = 0.309, p<0.001).

**Fig 2:**
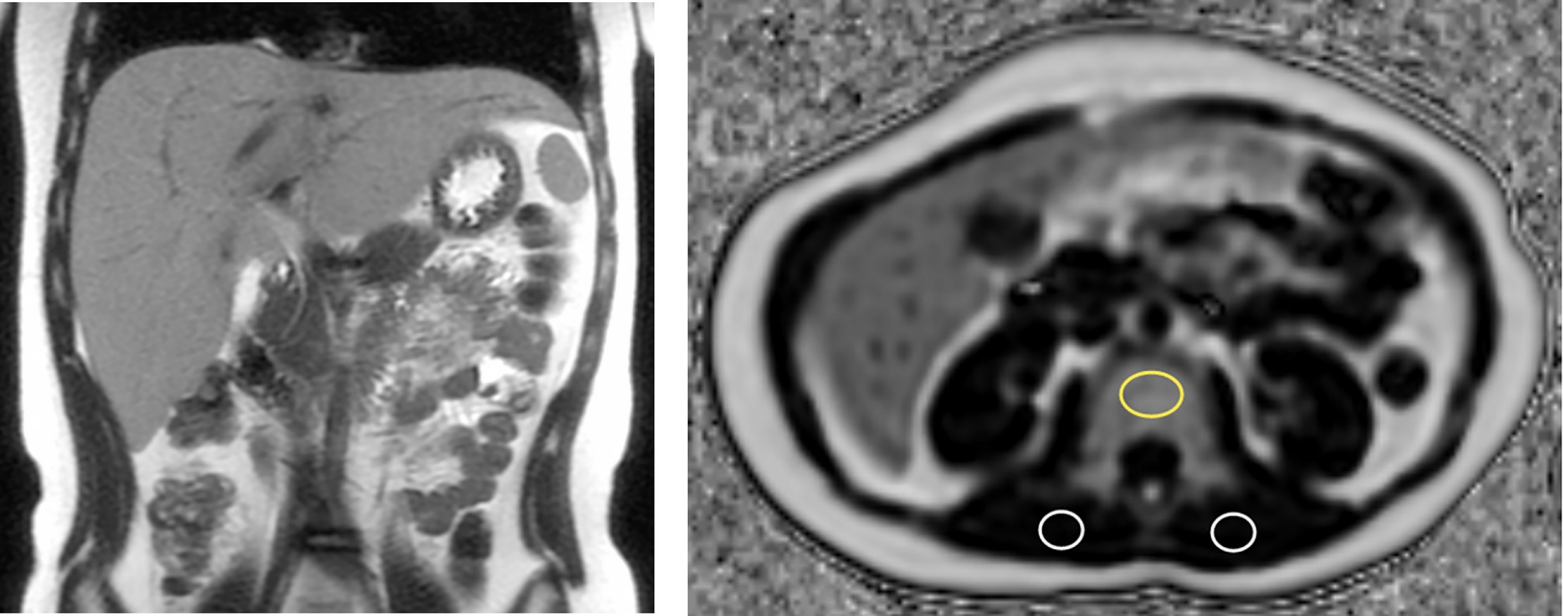
An 11-year-old boy with normal BMI, fatty liver, and high BM fat fraction. His height was 144 cm and weight was 40 kg. The BMI score was 19.3 kg/m^2^ and was within the normal range of his age group. (A) A coronal T2-weighted single shot fast spin echo image of the abdomen demonstrates hepatomegaly with diffusely increased T2 signal intensity of the liver from fat infiltration. The hepatic fat fraction was 48.0%. (B) An axial proton density fat fraction image of mid L2 level shows the location of regions of interest (ROIs) to measure vertebral BM fat fraction (yellow circle) and bilateral PSM fat fractions (white circles). The mean BM fat fraction was 50.6%, which was high; however, the mean PSM fat fraction was only 1.3%.

Table 1 shows the correlation results between the variables. Age, height, weight, and BMI were not correlated with liver or BM fat fractions. However, weight (ρ = 0.174, p = 0.035) and BMI (ρ = 0.247, p = 0.003) were positively correlated with PSM fat fraction (Fig 1A). The liver fat fraction showed a positive correlation with the BM fat fraction (ρ = 0.309, p<0.001) (Fig 1B) and PSM fat fraction (ρ = 0.171, p = 0.038) (Fig 2), even though the strength of correlation was poor to fair [21]. There was also a positive correlation between the BM and PSM fat fractions (ρ = 0.239, p = 0.004).

**Table 1:** Correlation between patient characteristics and fat fraction values.

	Liver fat fraction (%)		BM fat fraction (%)		PSM fat fraction (%)	
	ρ	p-value	ρ	p-value	ρ	p-value
**Age (years)**	-0.070	0.398	-0.084	0.314	0.013	0.877
**Height (m)**	-0.053	0.521	-0.053	0.521	0.089	0.286
**Weight (kg)**	0.009	0.915	-0.064	0.444	0.174	0.035
**BMI (kg/m^2^)**	0.114	0.169	-0.044	0.594	0.247	0.003
**Liver fat fraction (%)**			0.309	<0.001	0.171	0.038
**BM fat fraction (%)**					0.239	0.004

BM, bone marrow; PSM, paraspinal muscle; BMI, body mass index

Simple linear regression analysis demonstrated that BM fat had linear relationships with liver fat (β = 0.20, p<0.001) and PSM fat (β = 1.89, p = 0.004), but no relationship with age, sex or BMI group. Multiple regression analysis for BM fat also showed positive linear relationship with PSM fat (R^2^ = 0.198, Table 2). One percent increase of PSM fat was associated with 6.62% increase of BM fat in the normal BMI group (p = 0.009) and 0.82% (= 6.62–5.80) increase of BM fat in the obese BMI group (p = 0.027).

**Table 2:** Multiple regression for BM fat fraction in consideration with other variables.

Predictor		Estimate (β)	Standard error	p-value
**Age (years)**		-0.12	0.25	0.623
**Sex**	**Male**	Reference		
	**Female**	-1.12	1.64	0.496
**BMI group**	**Normal**	Reference		
	**Overweight**	0.31	6.52	0.962
	**Obese**	6.50	5.69	0.255
**Liver fat fraction (%)**		0.05	0.14	0.733
**PSM fat fraction (%)**		6.62	2.49	0.009
**BMI and liver fat fraction**	**Normal and liver fat fraction**	Reference		
	**Overweight and liver fat fraction**	0.13	0.17	0.447
	**Obese and liver fat fraction**	0.13	0.15	0.400
**BMI and PSM fat fraction**	**Normal and PSM fat fraction**	Reference		
	**Overweight and PSM fat fraction**	-3.01	2.84	0.290
	**Obese and PSM fat fraction**	-5.80	2.60	0.027

In partial correlation analysis, liver fat also showed positive correlation with BM fat fraction after adjusting for BMI (ρ = 0.316, p<0.001) and for both age and BMI (ρ = 0.309, p<0.001). Fat fractions of BM and PSM also showed positive correlations after adjusting for BMI (ρ = 0.258, p = 0.002) and for both age and BMI (ρ = 0.251, p = 0.002). However, liver fat and PSM fat showed no correlation after adjusting for BMI and/or age (Table 3).

**Table 3:** Subgroup results of fat fraction correlation analyses.

		Liver-BM		Liver-PSM		BM-PSM	
		ρ	p-value	ρ	p-value	ρ	p-value
**All patients**	**Without adjustment**	0.309	<0.001	0.171	0.038	0.239	0.004
	**Adjusting age**	0.304	<0.001	0.173	0.037	0.241	0.003
	**Adjusting BMI**	0.316	<0.001	0.149	0.073	0.258	0.002
	**Adjusting age and BMI**	0.309	<0.001	0.126	0.130	0.251	0.002
**BMI groups**	**Normal (n = 23)**	0.264	0.224	0.407	0.054	0.495	0.016
		(0.232)		(0.504)		(0.699)	
	**Overweight (n = 32)**	0.403	0.022	0.225	0.216	0.503	0.003
		(0.647)		(0.238)		(0.858)	
	**Obese (n = 92)**	0.278	0.007	0.068	0.520	0.129	0.222
		(0.773)		(0.099)		(0.233)	

BM, bone marrow; PSM, paraspinal muscle; BMI, body mass index

The numbers in parentheses mean power of correlation analysis from post-hoc power analysis.

In subgroup analysis based on BMI, liver fat and BM fat showed positive correlation in the overweight (ρ = 0.403, p = 0.022) and obese (ρ = 0.278, p = 0.007) groups but not in the normal BMI group (ρ = 0.264, p = 0.224). In the evaluation of power of correlation analysis (α = 0.05), normal BMI group had low power (0.232) compared with obese BMI group (power = 0.773) for analyzing the correlation between liver fat and BM fat, even though the correlation coefficients were similar (ρ = 0.264 in normal group and ρ = 0.278 in obese group). BM and PSM fat showed significant correlation in the normal BMI (ρ = 0.495, p = 0.016) and overweight (ρ = 0.503, p = 0.003) group, but not in the obese group (ρ = 0.129, p = 0.222). There was no correlation between liver fat and PSM fat in these subgroup analyses (Table 3).

## Discussion

There is a growing number of NAFLD patients worldwide, especially in patients with obesity. A recent study including 408 children demonstrated that NAFLD is common in children with obesity, found in nearly one-third of boys and one-fourth of girls [7]. Moreover, NAFLD is not only a liver disease, but is also associated with extrahepatic complications [9,10]. Therefore, it is necessary to distinguish the progression of NAFLD from normal growth in children and adolescents, but research on this topic is insufficient.

In this study, we investigated the changes of fat content in vertebral BM and PSM associated with age, BMI, and the degree of fatty liver in 147 pediatric patients with known or suspected NAFLD. We found that age and height were not associated with liver, BM, or PSM fat. BM fat increased according to increased liver fat, even after adjusting age and BMI. On the other hand, PSM fat was associated with obesity, but not with fatty liver. Therefore, in the progression of NAFLD in pediatric patients, fatty changes in the BM could be worse than normal, which may require further evaluation of BM metabolism. The accumulation of fat in skeletal muscle seems to be unrelated to NAFLD and associated with obesity.

Little is known about normal BM fat fraction in children. One previous report demonstrated vertebral BM fat fraction using a dual echo sequence in 13 healthy children as 39 ± 5% (range, 31–49%) [22]. In our study, only four patients were within normal limits of liver fat fraction and BMI, and their BM fat fraction was 28.2–52.0%, comparable with the previous study and was not different with other BMI groups.

There is growing interest in BM changes in obesity and NAFLD patients. In 2012, Pardee et al. [14] showed lower bone mineral density in obese children with NAFLD compared with obese children without liver disease. A recent meta-analysis confirmed that the presence and severity of NAFLD are associated with reduced bone mineral density in children and adolescents [23]. There could be a negative association between BM fat fraction and bone mineral density [24]. Therefore, our results of increased BM fat fraction in more severe fatty liver is consistent with previous results; however, there could be a confounding relationship between marrow adipose tissue and hematopoiesis, bone mineral density, bone strength, and metabolic function [25]. Additional study for the actual and detailed relationship between BM fat and bone mineral density in pediatric NAFLD patients is needed.

The one study that focused on BM fat content in pediatric NAFLD patients showed a positive correlation between lumbar BM fat fraction and liver fat fraction (ρ = 0.24, p = 0.008), even after adjusting for age, sex, and BMI [17]. However, the relationship was not found in girls, possibly due to the relatively small number of girls enrolled in the study (43 girls vs. 82 boys). The number of females (n = 35) was also much lower than that of males (n = 114) in our study, and the results of correlation between liver fat and BM fat fraction both before and after adjusting age and BMI (ρ = 0.309, p<0.001) was very similar as a consistent result.

BM fat distributes differently across age and skeletal site, and BM fat of the 4th lumbar vertebra showed a negative correlation with age (ρ = -0.36; p = 0.037) in 32 adolescents and young adults with obesity [16]. However, hematopoietic bone marrow of the axial skeleton and lumbar spine generally persists with minimal fatty conversion before the age of 20 in healthy adolescents [26]. Our study also demonstrated no relationship between BM fat and age or height in children. Although the study does not provide a normal BM fat fraction according to childhood age, it appears that in pediatric patients, increased BM fat should be considered a pathological finding, and not a function of normal growth.

As a metabolic disorder, the pathogenesis of NAFLD has complex interactions among hormonal, nutritional, and genetic factors [27]. Since there is a report that demonstrated sucrose overfeeding increased liver and muscle fat deposition [28], there may be more fat deposits in the muscles of NAFLD patients than in that of healthy individuals. However, there are only reports about the relationship between aging and muscular fatty infiltration in adults in the literature [29,30] and little is known about muscle fat in children with NAFLD. Only one magnetic resonance spectroscopy study performed in Danish children demonstrated that fat deposit in liver and muscle was associated with obesity-related metabolic dysfunctions, which could increase cardiovascular disease risk [31]. Our study showed a positive correlation between obesity and muscle fat infiltration in PSM regardless of growth in children with NAFLD. Although the risk of cardiovascular disease has not been investigated in this study, it is thought that early diagnosis of pathologic muscle fat depositions and reduction through appropriate treatment will be important for the prevention of long-term cardiovascular complications, as fat deposits in pediatric patients can be reduced reversibly through multidisciplinary intervention programs [32]. Further prospective research is needed for this topic.

We acknowledge some limitations of our study. First, it was a retrospective study and we included only patients with fat quantification MRI. As such, there were a limited number of normal group patients with normal hepatic fat content and normal BMI. The second limitation is that it was a cross-sectional study and we could not demonstrate causality of the results. The third limitation is the limited area of BM or PSM fat evaluation in our study. It is necessary to verify whether the region in which we measured the fat deposit in BM and PSM accurately reflects the state of the whole body. Additional study is needed for this issue.

## Conclusions

The fat fraction of BM or PSM is not associated with growth in children with NAFLD. The severity of fatty liver is associated with increased vertebral BM fat. The severity of obesity has a positive relationship with skeletal muscle fat infiltration but is not associated with fatty liver. It will be necessary to set up different focusing sites of clinical concern depending on the status of obesity and NAFLD during growth in children.

## Supporting information

S1 Data (XLSX)
